# Feasibility of video recording interpersonal interactions between patients and hospital staff during usual care

**DOI:** 10.1186/s40814-022-01052-w

**Published:** 2022-04-29

**Authors:** Angela L. Todd, Lynette Roberts, Kirsty Foster

**Affiliations:** 1 Women and Babies Research, Northern Clinical School, Faculty of Medicine and Health, The University of Sydney, The Douglas Building, Royal North Shore Hospital, St Leonards, NSW 2065 Australia; 2 Northern Sydney Local Health District, The Kolling Institute, St Leonards, NSW 2065 Australia; 3 Academy for Medical Education, University of Queensland, Herston, QLD 4006 Australia; 4 Northern Clinical School, Faculty of Medicine and Health, The University of Sydney, St Leonards, NSW 2065 Australia

**Keywords:** Interpersonal communication, Patient experience, Quality improvement, Video recording, VRE, Feasibility

## Abstract

**Background:**

Video-reflexive ethnography (VRE) has been used to record aspects of patient care which are then shared with staff to drive self-identified improvements. Interpersonal interactions between patients and hospital staff are key to high-quality, patient-centred care and mostly occur randomly throughout a patient’s hospital stay. One of the most common types of hospital admission is for women giving birth.

**Aims:**

To assess the feasibility of adapting the VRE methodology to capture naturally occurring interactions between patients and health staff over an extended period during hospital admission, and to assess whether the approach would yield useful interaction data.

**Participants:**

Twelve women, who had a planned caesarean section at 37+ weeks, were considered low risk (no known medical or obstetric complication) and were admitted to a postnatal unit after giving birth, and the staff who attended them.

**Methods:**

This study took place in a large hospital in Sydney, Australia, where approximately 2200 women give birth each year. Continuous unattended video recordings were made during each woman’s hospital stay to capture interactions with hospital staff. The recordings were reviewed to determine what kinds of interaction data could be obtained.

**Results:**

In order to recruit 12 eligible women, we needed to invite 45 to participate. The estimated recruitment period of 3–4 months had to be extended to 8 months. A fixed video camera was successfully installed in the hospital room of each woman and a remote control provided. A total of 246.5 h of video recordings was obtained, of which 38 h (15.5%) involved interpersonal interactions with staff. Two women reported negative responses from staff about being video recorded. Both quantitative and qualitative data could be obtained from the recordings.

**Conclusion:**

Video recordings of interpersonal interactions between patients and staff in an in patient hospital care setting can be obtained and can provide unique insights into the complexity of healthcare delivery. However, significant contextual barriers can exist to engaging staff in quality improvement initiatives that are not part of their usual healthcare activities.

## Key messages regarding feasibility

- Video-reflexive ethnography (VRE) is usually used to record specific aspects of patient care; however, interpersonal interactions between patients and healthcare staff during a hospital stay can occur at random times and require innovative study methods.
- Continuous video recordings can capture patient-staff interactions as they naturally occur and provide rich information about the quality of care.
- Multiple strategies are needed to support the engagement of busy healthcare staff in health services research.

## Introduction

Good interpersonal communication is essential to the development of effective relationships between clinicians and patients and is associated with high-quality care, positive patient experiences and improved health outcomes [1–3]. In healthcare, it has been suggested that effective interpersonal communication skills support task-focused exchanges such as obtaining a medical history, explaining a medical procedure, or giving therapeutic information, as well as interactions that are more relational and patient-centred and serve to foster a trusting and humanistic relationship [4]. Interpersonal skills such as demonstrating empathy, being responsive and adaptive to individual needs and circumstances, showing caring, kindness, understanding and trust have been shown to contribute to building therapeutic relationships between patients and healthcare providers [2–4]. Patients who report poor interpersonal interactions with health staff are more likely to be dissatisfied with their healthcare experiences, lodge complaints and take legal action [5].

Health services research investigates how social factors, financing systems, organisational structures and processes, health technologies and personal behaviours affect healthcare quality and delivery including equity of access, cost and impacts on health and well-being [6]. A key objective of health services research is to understand and improve healthcare. The complexity and dynamics in healthcare settings can make reductionist research methods, commonly used to focus investigations on specific variables, not only difficult to apply but insufficient to be impactful especially in busy workplaces. Video-reflexive ethnography (VRE) is a methodology that has been used to drive improvements through recording aspects of patient care in situ [7, 8]. VRE provides a means of capturing complex clinical situations in context and has been shown to support positive changes in a range of clinical settings including infection control, dementia care, end-of-life care and patient-centred care [9–13]. To date, most studies that have used VRE for healthcare improvement have focused on specific behaviours or issues, for example, communication between senior and junior clinicians during handover [11], mother-infant skin-to-skin contact following birth [14] or dementia patients’ views about their hospital environment [13]. However, a recent study used video recording to observe interactions on multiple occasions between healthcare staff and parents, as well as between health professionals during pregnancy, childbirth and after birth. The recordings showed that both formal and informal strategies facilitated communication and collaboration between individuals [15]. In most VRE studies, a researcher positions him/herself unobtrusively in the clinical setting with a small camera to record target behaviours. Short video extracts (snippets) are subsequently selected and shown to participating staff and/or patients. These snippets reproduce the dynamics and complexity of everyday practice and reveal behaviours that can help or hinder quality care, but which are often not noticed at the time. Discussion and self-reflection are encouraged among participants to help make personal and collective decisions about change [16].

Our feasibility study explored whether we could adapt the VRE methodology to capture, in a more holistic, authentic way, interpersonal interactions that occur between patients and staff over an extended period during a hospital admission [17]. We also assessed whether patients and staff would accept being filmed, and if this approach could yield useful video data. Pregnancy and childbirth account for a high proportion of hospital admissions each year [18]. Most pregnant women are young, healthy and admitted to hospital for short periods [19]. For many women, the birth admission is their first experience with hospital-based care, potentially influencing future engagement with health services for both women and their families [20]. This study focused on interactions that occurred between women and hospital staff after the women had given birth and been transferred to a postnatal unit. Previous research has shown opportunities for improving women’s interpersonal and care experiences particularly after birth [21]. We included women who had had a planned caesarean section since they have regular post-operative care from hospital staff, providing multiple opportunities to capture interactions. Rather than having a researcher present to record specific behaviours, we installed a camera in the woman’s hospital room to allow continuous recording over several days. The camera remote control was given to the woman to provide the option of privacy.

In this study, we addressed the following questions:

- Will women and staff agree to extended periods of video recording?
- Can the VRE methodology be incorporated into an inpatient maternity care setting?
- Can the unattended video recordings provide useful information about interpersonal interactions between women and staff in a postnatal unit?

The feasibility study results would help determine whether the modified VRE methodology could be used in a larger future study to review interpersonal interactions between patients and hospital staff. We considered feasibility would be supported if we could demonstrate that (i) patients and hospital staff were willing to be video recorded, (ii) an unattended video camera could be safely and easily installed into the hospital setting and (iii) useful data could be collected about naturally occurring interpersonal interactions between patients and hospital staff.

## Methods

This study was a small cross-sectional observational study.

### Setting

The hospital in this study is a large public teaching hospital in Sydney, Australia, with approximately 80,000 patient admissions annually including 2200 women who give birth.

### Engaging health leaders

Early efforts were made to engage with senior staff to plan the project, recognising potential sensitivities around the use of video to record patient-staff interactions. Initial meetings were held with the Directors of Maternity Care (medical and midwifery) and the managers of the Antenatal Clinic, Birth Unit and Postnatal Unit. While the focus of the study was on interactions during postnatal care, many staff work across all three units. The Directors supported the project as a quality improvement initiative that aimed to explore the interpersonal care experiences of women following birth.

### Participants

The participants in the study were women meeting the eligibility criteria (described below) who consented to have a video camera in their hospital room, and all medical, midwifery, administrative and catering staff attending the Postnatal Unit during the study period.

#### Women

Pregnant women aged between 18 and 45 years who were booked to have a planned caesarean section at 37+ weeks and were considered low risk (no known medical or obstetric complication) were eligible to participate. Women were invited to participate at the hospital’s Antenatal Clinic by a research midwife (not involved in providing clinical care), usually around 35–36 weeks’ gestation. The planned nature of the birth allowed the research midwives to recruit women in advance, confirm their willingness to participate at the time of the birth admission and install the video camera on their arrival in the Postnatal Unit (after giving birth). Women who consented to participate but who subsequently developed a complication (i.e. necessitating early/emergency delivery) or who had spontaneous labour before the date of the planned caesarean section were withdrawn from the study. Women themselves could withdraw from the study at any time and/or request deletion of video data. We aimed to recruit 12 women for this feasibility study, deemed an adequate number for pilot and early studies when no prior information is available for estimating sample size [22]. In consultation with clinical staff, we estimated recruiting 12 women would take 3–4 months and provide a diversity of women. We appreciated that some women would not want to have a video recorder operating in their room during the first days after giving birth—a time that is very special to most families.

#### Staff

All staff who worked in the hospital’s Antenatal Clinic, Birthing Unit or Postnatal Unit were considered participants in the study since a high proportion of staff work in more than one location, either on rotation or when needed. Staff were invited to attend one of several information sessions about the study, held at different times of day over a period of 6 weeks prior to study commencement. Information was also circulated by lead clinical staff to their teams by email, and hard copy information sheets were circulated in the Antenatal Clinic, Birthing Unit and Postnatal Unit. Meetings were held with catering staff supervisors and written information provided. Staff were advised that the aim of the project was to help provide high-quality care by exploring interpersonal interactions with patients. It was understood that some staff might feel uncomfortable or nervous about being video recorded, and staff were assured that any video data involving them would only be shown to other staff with their permission. The local Human Research and Ethics Committee which approved this study accepted the use of implied consent from staff for video recording unless they opted out in writing (HREC/16/HAWKE/128). Video footage of staff who opted out but who were inadvertently filmed would be deleted. Any staff member who was video recorded could also subsequently request deletion of video data pertaining to them.

### Video recording process

The Postnatal Unit in this study endeavours to provide single-occupancy rooms for women who have had a caesarean section. A research midwife monitored the time of birth for each participating woman and, shortly before transfer from Operating Theatre to the Postnatal Unit, a lightweight video camera was mounted on the wall behind the bed in her room. This location was chosen in consultation with the unit manager and was visible to anyone entering the room. A clock above the door in each room was captured in the filming frame, allowing identification of time of day and length of time recorded. A notice was also placed on the outside of the door of the room advising that video recording was underway.

After the woman arrived in her room, a research midwife explained the recording procedure, the operation of the video remote control and the process of daily collection and replacement of the video data card. Each woman had the option of stopping and re-starting the video camera herself to allow for times of privacy. A researcher was not present during the filming process. Women were assured that any filming of family or friends would be ignored since the focus of the study was interactions with hospital staff. Women were encouraged to record on camera any comments about their interactions with staff.

Feedback was obtained from the research midwives about the processes of recruiting women to the study (and reasons for declining), and video recording in the Postnatal Unit including logistics and any issues raised by women or staff. The medical educator/facilitator who led all information sessions at the commencement of the project and feedback sessions with staff to discuss selected video snippets (KF) also kept field notes.

### Video recording analysis

The video recordings were viewed independently by at least two members of the research team to identify sections that captured interactions between the women and staff. These sections were then assessed to determine the nature of the recorded interactions and were coded thematically. We noted the length of the interaction time (minutes/seconds) and which types of staff were involved (midwife, doctor, catering/meals staff, other (e.g. physiotherapist, administrative)). The video analysis took place within an interpretivist paradigm exploring the nature of interactions from a sociocultural perspective [23]. Short video ‘snippets’ suitable for reflective feedback sessions with staff were selected. Staff in the Postnatal Unit were invited to attend feedback sessions to view and discuss different video snippets. Prior to showing any snippet to a group, the staff involved viewed the snippet privately and gave permission for its use. The feedback sessions were conducted by the medical educator/facilitator (KF).

## Results

### Will women and staff agree to extended periods of video recording?

#### Women

**Fig. 1:**
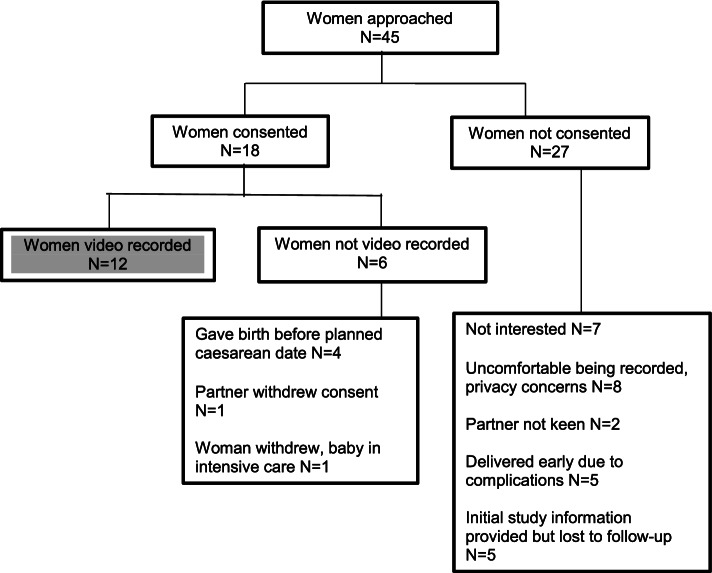
Study participant recruitment

To achieve our recruitment target of 12 participants, we invited 45 pregnant women to participate over 8 months (April–December 2017). Eighteen women consented; however, the risk status of 4 changed after giving consent (from low to higher risk due to emerging complications) and 2 withdrew, leaving 12 (27%) women who progressed to video recording (see Fig. 1). These changes were identified by the research midwives who monitored the consenting women as they progressed during their pregnancies. We did not formally collect demographic or clinical information about the participants in the study. Comments made to the recruiting midwives indicated these women were enthusiastic about the study although some partners were less keen. Among the 33 women who were invited but did not participate, 8 women or their partners raised specific concerns about being video recorded or mentioned issues of modesty and privacy.

#### Staff

The Postnatal Unit Manager was actively engaged throughout the planning and implementation of the project, attending meetings, rescheduling staff to attend information sessions, assisting with project communications, etc. However, we failed to adequately engage the Antenatal Clinic and Birth Unit Managers despite multiple attempts.

Fifteen information sessions were held to introduce and describe the study, attended by more than one hundred staff who could attend one or more sessions. During these sessions, staff expressed a range of views about the project: some were enthusiastic, some questioned whether the presence of a video camera would elicit ‘better’ communication behaviours than usual care (Hawthorn effect) and some were suspicious that the recordings would be used by senior management to monitor clinical practices. Staff were assured that senior management had no access to the recordings except for selected snippets which focused on interpersonal interactions, and which would be approved for sharing by the staff involved.

Over the 8-month recruiting and recording period, different junior doctors and midwifery and nursing students rotated through the Postnatal Unit, and a range of casual and agency staff were employed, often on short-term contracts, requiring multiple information sessions. Internal communication with staff about the project was inconsistent over time. As a result, some staff felt unaware or inadequately informed about the project. This included staff who only worked night shifts, even though we scheduled some information sessions to complement the start or end times for these shifts. Further, while some doctors attended the initial information sessions, they did not otherwise engage with the study despite multiple invitations and explicit support for the project by senior medical staff.

### Can the VRE methodology be incorporated into an inpatient maternity care setting?

The location of the video camera on the wall behind the woman’s bed in the Postnatal Unit gave a clear view of people entering the room and the area around the woman’s bed but not the woman’s face (unless she left the bed). Alternate positions for the camera were considered to record facial expressions during interpersonal interactions, for example, by fixing the camera to other standard equipment in the room or on a tripod, but these options breached health and safety regulations. The length of recording time also required access to a fixed power supply, which influenced the location of the camera. Night-time filming appeared ‘ghost-like’ on video, reducing the quality of visual information but audio information was clear.

While each woman was given a remote control and the option of switching the camera on/off, 9 of the 12 women chose continuous recording. Recorded time ranged from 2 h 39 min to 38 h 42 min per woman (mean 20 h 33 min). The camera stopped when the video card was full, or a research midwife came for the daily check and video card replacement. Each video card stored about 12–14 h of recordings. Efforts to capture weekend stays in hospital proved unsuccessful; in the busyness of the unit’s usual activities and with more limited staffing, the task of changing the video card was forgotten.

We had invited the participating women to record on camera any reflections they might have about their interactions with staff in the Postnatal Unit. Three women reported being very happy with the care they received and thanked the staff on camera shortly before leaving hospital. Two women reported to a research midwife that they had had a negative encounter with a midwife who had told them they were opposed to being video recorded and/or had no knowledge about the study. These staff insisted that the camera be switched off. One of the women reported on camera that she felt very distressed by the encounter and the midwife did not attend her room again.

### Can the unattended video recordings provide useful information about interpersonal interactions between women and staff in a postnatal unit?

Viewing and coding the video recordings were highly labour-intensive. The total recorded time for the 12 women was 246 h 34 min. Only 38 h (15.5%) of the recorded time involved interpersonal interactions between the women and staff; for the remaining time, the women were alone or with family and friends. Attempts to fast-forward through footage that seemed uneventful (e.g. while the woman was sleeping or had visitors) were not effective, as it was easy to miss brief visits from staff. The recorded interactions did not require transcribing, as the VRE method uses video segments to demonstrate behaviour in context as the teaching and reflection tool. The most frequent staff interactions were with midwives (total recorded time 33 h 29 min), with total recorded times ranging from 55 min to 5 h 7 min. (For the purposes of coding, the group “midwives” included nurses or midwifery trainees attending the women since the distinction between these roles was usually not known to the women.) Video recordings of interactions with doctors were mostly brief and infrequent (total recorded time 2 h 51 min). Obstetricians and anaesthetists conducted post-operative checks, or sometimes a paediatrician attended to check the baby. Catering staff attended the women several times each day to bring food for breakfast, lunch and dinner as well as morning and afternoon tea. While these visits were regular, they were fleeting. Ad hoc visits from other staff were also recorded, such as physiotherapists, clerical staff, lactation consultants and a chaplain (total time 1 h 37 min).

The video recordings demonstrated the diverse and individual circumstances and needs of the 12 participating women. For example, Woman #1 and her partner were South American, and English was not their first language. Family members and friends visited the woman during her hospital stay and were seen on video to assist her in many ways (e.g. cutting up food, moving the baby’s crib closer to the bed, picking up the baby when crying, changing a nappy, helping the woman prepare for a shower, etc.). This was the woman’s third child and she appeared relaxed and confident in managing her baby especially with her family around her but was experiencing considerable discomfort following the caesarean section. In contrast, Woman #3 had little family support (most of the extended family lived in the UK) and was quite anxious about her recovery and breastfeeding her baby. Woman #8 appeared self-assured, confident and keen to manage herself and her baby as much as possible. Because of the differences in individual women’s needs and contexts, the interactions with maternity staff were never the same.

While recognising the diversity between patients, we were able to identify five common types of interactions: conversations, collaborations, staff noticing care needs and nurturing, staff informing and instructing patients and non-verbal communication (Table 1). We also found examples of both positive and less positive interaction ‘snippets’. While some staff referred to the presence of the camera, the recorded interactions appeared authentic rather than staff ‘performing at their best’. Obtaining approval from selected staff for the use of snippets involving them proved challenging: screenshots had to be taken, the staff member identified by the Unit Manager and contact details provided to the researcher to arrange viewing of the snippet. Some staff in the selected snippets no longer worked in the Unit or had been casual staff not known to the Unit Manager.

**Table 1:** Analysis of interactions between women and staff—main themes and examples of associated interactions

Theme	Examples of interactions
Conversations	Getting to know each other, midwife and woman connecting personally, building rapport and trust, midwife asking about the family (e.g. ‘how old is your other child?’), making jokes, compliments (e.g. ‘such a beautiful baby’)
Collaborations	Working together as a team, midwife changing the bed linen while the woman rolled from one side of the bed to the other, helping the woman get up for a shower
Noticing needs and nurturing	Physical comfort, midwife adjusting tight compression stockings, lowering room lighting, adjusting room temperature, changing soaked bed, providing extra pillow, helping the woman to sit up, ‘call me/buzz me anytime/whenever you need anything’
Informing and instructing	Giving relevant information and instruction, breastfeeding help, details about the procedure for removing the catheter or cannula, details about physiotherapy and lactation services at the hospital
Non-verbal communication	Encouragement and positive feedback, especially during breastfeeding, midwife smiling, nodding, showing extended eye contact, physical proximity

Ten face-to-face feedback sessions were conducted, attended by 69 staff, to share and discuss video snippets of interactions between women and staff. The sessions were limited to 30 min, scheduled at the unit’s regular professional development times, to facilitate staff attendance. The snippets engaged staff and elicited discussion about the interactions. Several staff remarked that they rarely had the chance to observe how colleagues interacted with women and how this contrasted with their own behaviour.

## Discussion

A feasibility study is designed to focus on process and answer the question “Can it work?” [17]. In this feasibility study, we assessed whether a modified version of the VRE methodology, which included an unattended, fixed camera, could be used to make extended video recordings over several days of naturally occurring interpersonal interactions between maternity patients and the hospital staff who attended them. The patients in this study were women who had given birth by planned caesarean section and were receiving care in a postnatal unit. While most women giving birth are generally healthy and well, women having a caesarean section need to receive care that is similar to other surgical patients, including regular clinical monitoring post-surgery, pain management, assistance with toileting and information about recovery and progress. Like other patients, there was considerable diversity between the women and staff in our study, but we also identified common types of interpersonal interactions. We would suggest that the five types of interactions (conversations; collaborations; staff noticing care needs and nurturing; staff informing and instructing patients; and non-verbal communication) are likely to be relevant to many hospital patients.

We identified three criteria that would lend support to the use of this modified VRE methodology in a larger study. The first was the willingness of patients and hospital staff to participate and be video recorded. Our results suggested enthusiasm among some women and staff, but also disinterest and even opposition among others. Many women who were invited to participate in this study declined, often because they were either not interested in the study or for privacy and modesty reasons. In contrast, the women who did consent appeared comfortable about being recorded and perhaps more self-confident and self-assured. Although we recognise that our participants may not be representative of all women giving birth or all women having caesareans, we would suggest that the interactions between the women and staff appeared typical for this population. It was the nature of these interactions which was the focus of this study. Various staff expressed early concerns about being filmed, raising issues of feeling self-conscious or being monitored by managers. It also became apparent that many staff did not appreciate the potential value of the study to facilitate improvement in the quality of care. Patients can feel quite vulnerable when in hospital, and staff can feel threatened or suspicious about being filmed [24, 25]. Such feelings may have contributed to the relatively low consent rate among women in our study, and some of the negative responses from staff both before and during the filming phase of the study. In an attempt to manage such responses, some studies using VRE have embedded researchers among clinical staff for a period of time to develop trust before recording their behaviour [8]. Interestingly, when we viewed the video recordings, the presence of the camera was often mentioned but appeared to be quickly ignored; the interactions between the women and staff seemed genuine and included examples of very positive as well as some less ideal exchanges.

The clinical environment in which this project was embedded is a 35-bed postnatal unit in a large urban teaching hospital providing care to over 2200 birthing women, and approximately 80,000 patient admissions each year. While the project team worked with the manager of the unit, the project appeared to be viewed by staff as separate from and outside of their work rather than integral to it. Although we sought to engage all senior managers, we had only partial success. Additional champions and peer leaders might have helped achieve greater buy-in from staff, particularly the medical staff who proved difficult to engage. The focus of the project on interpersonal interactions with patients also did not seem to appeal to medical staff as we had anticipated, and perhaps even threatened some. We sought to inform as many staff as possible about the project prior to commencement, but some remained suspicious of being filmed, perhaps pointing to wider issues between staff and managers. We also acknowledge that the negative reactions from a small number of staff during filming may have been due, at least in part, to not being well-informed about the study, possibly due to the extended recruitment period, because they had joined the unit after the initial study briefings, or were casual agency staff or night staff. Although we were aware of only two staff objecting to being filmed, we had not foreseen that they might verbally ‘attack’ or upset women.

The second feasibility criterion we considered focused on the practicalities of embedding an unattended video-camera over an extended period in a hospital setting. To a large extent, this was successfully achieved. Most other VRE studies have typically focused on discrete behaviours and exchanges where timing is more predictable, and a person (usually researcher) operates the video recorder and moves around. The presence of such a person can impact the observed behaviour [24] and can elicit inadvertent interactions between the study participants and the camera operator [26]. We avoided these confounding effects by mounting the camera in a fixed position in the patient’s room, which influenced the type of camera and its location. To allow continuous filming, we needed access to a power source and daily exchange of the data storage card. Although we gave women a remote control to switch the video recorder off and on, few of them used it and instead allowed the camera to operate until the data card was full. These logistical issues were manageable. Nonetheless, the fixed position of the camera, while a pragmatic solution for the extended recording time, meant that some important aspects of interactions between the women and staff were not well captured, for example, facial and other non-verbal expressions by the women, and when staff were in the room but out of range of the camera.

The third criterion we examined for feasibility with the modified VRE methodology was whether it would yield useful data about interpersonal interactions between patients and hospital staff. A significant benefit of VRE is that it allows us to view complex clinical situations in context and identify potential opportunities for improved care practices [8, 11]. Interpersonal interactions are a complex interplay of verbal and non-verbal behaviours, as well as contextual factors such as the physical environment, the personalities of the individuals involved and the relationship that exists between them. In this study, the information captured on video about interpersonal interactions between patients and staff provided a literal ‘gold mine’ of data about the many diverse, behavioural components that make up exchanges between people. Nonetheless, in nearly 250 h of video recordings, only 16% of the time involved interpersonal interactions between women and staff. Importantly, these comparatively brief encounters with staff play a significant role in patients’ care experiences [21] and have been linked with patient safety and clinical effectiveness [27]. They may be short, but they are impactful.

VRE has previously been used in a range of clinical settings, including maternity care. Stevens and colleagues [14] used VRE to study the barriers to skin-to-skin contact between mothers and newborns after birth by caesarean section. The video recordings showed that different health professionals attended to different ‘parts’ of the mother-baby dyad, with obstetricians ‘owning’ the lower half of women, anaesthetists ‘owning’ the top half and midwives ‘owning’ the baby. These contextual and nuanced dynamics were made visible by VRE and would be difficult to discern by other research methods. Another maternity care study using VRE by Korstjens et al. focused on communication and collaboration behaviours during pregnancy, birth, and after birth between parents and health professionals [15]. In addition to formal ‘known’ mechanisms designed to support effective communication, such as protocols, procedures and meetings, the video recordings helped uncover informal strategies such as engaging in small talk or humour, or non-verbal behaviours, which were often unnoticed or disregarded but significant for facilitating connectedness and ‘being together’. Our video recordings also demonstrated the many and different formal and informal interactions that hospital staff have with women recovering post-surgery and caring for a newborn, particularly with midwives who seamlessly weaved clinical procedures and instructions with informal ‘chatting’ and shared laughs. In contrast to the findings of Stevens et al., we found that the staff in our study were, in the main, woman-centred in their interactions. Midwives, and a senior doctor who chose to spend time with a woman, displayed many informal ways of building connections and trust within the therapeutic relationship, akin to the strategies described by Korstjens and colleagues. Our own work and these other VRE studies demonstrate the power of video recordings to reveal the many complexities in interpersonal exchanges between patients and health professionals,

## Conclusions

VRE has been used successfully in a range of clinical settings to drive improvements in healthcare delivery. This feasibility study found that it was possible to adapt VRE to record ‘real-world’ interpersonal interactions over an extended period between women who had recently had a caesarean section and hospital staff in a postnatal unit. Further, this approach generated a significant amount of video data that would likely be useful for further analysis. We identified five common types of interactions that could inform future research in other inpatient settings about the provision of healthcare. However, we identified several ‘human’ challenges during this feasibility study that bear consideration. Some patients and staff are uncomfortable about being video recorded. This needs to be acknowledged and managed as part of participant recruitment planning in any future research using VRE. Perhaps more fundamentally, effectively engaging busy health staff in a research project can pose difficulties. In this study, some senior managers provided high level support, and one unit manager was actively engaged, but otherwise for most staff the project seemed of peripheral interest, or if they were interested, their clinical work demands limited their ability to be involved. We believe these practical issues are not unique to maternity units or Australian hospitals or the use of VRE, and significantly curtail truly collaborative health services research and its translation. Research needs to be embedded as an essential element in safe, high-quality healthcare and to be part of the role of health professionals.

## Data Availability

The collected video data and study materials are safely stored on a university server to protect the participants’ identities.
